# Correction to: Respiratory morbidity in preschool and school-age children born very preterm and its association with parents’ health-related quality of life and family functioning

**DOI:** 10.1007/s00431-023-04829-0

**Published:** 2023-01-24

**Authors:** Gabriela P. Peralta, Raffaela Piatti, Sarah R. Haile, Mark Adams, Dirk Bassler, Alexander Moeller, Giancarlo Natalucci, Susi Kriemler

**Affiliations:** 1 Epidemiology, Biostatistics and Prevention Institute (EBPI), University of Zurich, Zurich, Switzerland; 2 University of Zurich, Zurich, Switzerland; 3 Newborn Research, Department of Neonatology, University of Zurich and University Hospital Zurich, Zurich, Switzerland; 4 Department of Respiratory Medicine and Childhood Research Center, University Children’s Hospital Zurich, Zurich, Switzerland; 5 Larsson-Rosenquist Centre for Neurodevelopment, Growth and Nutrition of the Newborn, Department of Neonatology, University of Zurich and University Hospital Zurich, Zurich, Switzerland

**Correction to: European Journal of Pediatrics** **https://doi.org/10.1007/s00431-022-04783-3**

The original published version of the above article contained an error. The abstract section contained unnecessary data; these data are not part of the content. The text highlighted in bold should have been deleted.

The purpose **[CMC1]** of this study is to describe **[CMC2]** the prevalence and severity of respiratory symptoms in children born very preterm and to assess their association with parents’ health-related quality of life (HRQoL) and family functioning. We conducted a cross-sectional study and recruited children born less than 32 weeks’ gestation between January 2006 and December 2019, in the greater Zurich area, Switzerland. Between May and December 2021, parents were invited to complete an online survey for their preterm child and for a control term born (≥ 37 weeks’ gestation) sibling aged 1 to 18 years. We used a validated questionnaire to assess respiratory symptoms and thePediatrics Quality of Life Family Impact Module (PedsQL FIM) to assess parents’ HRQoL and family functioning. The survey was completed for 616 very preterm children (99 with bronchopulmonary dysplasia (BPD)) and 180 controls. Girls made up 45% (46% in controls) of the sample, and 63% (60% in controls) of participants were aged 6 to 18 years (school-age). Very preterm children reported a higher risk of respiratory symptoms than controls, especially preschoolers and those with moderate-to-severe BPD. Parents of children with “mild” and “moderate-severe” respiratory symptoms had on average − 3.9 (95%CI: − 6.6 to − 1.1) and − 8.2 (− 11.2 to − 5.2) lower PedsQL FIM total score, respectively, than parents of children with no symptoms. The same pattern was observed after stratifying by age categories. **[CMC1]LE: Please check if the edit to the sentence “The purpose of this study is to…” retained its intended meaning. Otherwise, please amend. [CMC2]LE: As per journal style, abstract should be unstructured."**

*Conclusions:* Our study suggests that respiratory morbidity in very preterm children has a negative impact on parents’ HRQoL and family functioning, even beyond the first years of life.

The original article has been corrected.

